# Epidemic Cholera

**Published:** 1847-12

**Authors:** 


### EPIDEMIC CHOLERA.

It seems now quite certain that this pestilence is again advancing westwardly; and that, too, nearly in the path it traveled before. It has actually entered Europe, by the eastern edge, and will no doubt hold its way to and through the Atlantic kingdoms of the continent; thence to invade America. Should it reach Ireland, before the destitute people of that country have recovered from the famine they have just passed through, its ravages would doubtless prove appalling. In our next number we propose to give a condensed summary of the facts indicating its present march.

